# The Role of the Heat Shock Cognate Protein 70 Genes in Sex Determination and Differentiation of Chinese Tongue Sole (*Cynoglossus semilaevis*)

**DOI:** 10.3390/ijms24043761

**Published:** 2023-02-13

**Authors:** Qian Liu, Yue Wang, Leilei Tan, Wenxiu Ma, Xiaona Zhao, Changwei Shao, Qian Wang

**Affiliations:** 1 College of Fisheries and Life Science, Shanghai Ocean University, Shanghai 201306, China; 2 National Key Laboratory of Mariculture Biobreeding and Sustainable Goods, Yellow Sea Fisheries Re-search Institute, Chinese Academy of Fishery Sciences, Qingdao 266071, China; 3 Jiangsu Key Laboratory of Marine Biological Resources and Environment/Jiangsu Key Laboratory of Marine Biotechnology, Jiangsu Ocean University, Lianyungang 222000, China; 4 Laboratory for Marine Fisheries Science and Food Production Processes, Pilot National Laboratory for Marine Science and Technology (Qingdao), Qingdao 266237, China

**Keywords:** *hsc70* genes, high temperature, sex determination and differentiation, *Cynoglossus semilaevis*

## Abstract

Fish sex determination can be affected by environmental temperature. This process relies on temperature-sensitive proteins such as heat shock proteins (HSPs). Our previous work found that heat shock cognate proteins (HSCs) may participate in high-temperature associated sex reversal of Chinese tongue sole (*Cynoglossus semilaevis*). However, the role of *hsc* genes in responding to high temperature and affecting sex determination/differentiation remains unclear. Here, by using *C. semilaevis* as model, we identified *hsc70* and *hsc70-like*. *hsc70* was abundant in the gonads with a testicular-higher expression at all gonadal development stages except for 6 months post fertilization (mpf). Intriguingly, *hsc70-like* showed higher expression in testes from 6 mpf on. Both long-term heat treatment during the temperature-sensitive sex-determining period and short-term heat stress at the end of this period caused different expression of *hsc70*/*hsc70-like* between sexes. The dual-luciferase assay results also suggested that these genes can respond to high temperature rapidly *in vitro*. Heat treatment of *C. semilaevis* testis cells overexpressed with *hsc70*/*hsc70-like* could affect the expression of sex-related genes *sox9a* and *cyp19a1a*. Our results indicated that *hsc70* and *hsc70-like* were key regulators linking external high-temperature signals with sex differentiation *in vivo* and provide a new idea for understanding the mechanism by which high temperature affects sex determination/differentiation in teleosts.

## 1. Introduction

In vertebrates, the structure of the gonads is highly conserved [1], while sex is affected by a wide range of mechanisms, mainly subdivided into genetic sex determination (GSD), environmental sex determination (ESD), and the combined genetic–environment interaction [2]. A number of factors can influence sex in an ESD system, including temperature, light, and social stress [2,3]. However, in lower vertebrates such as fish, GSD is often influenced by environmental factors, with the most important factor being temperature [4]. Therefore, in vertebrates, some external environmental factors may participate in sex determination and differentiation, which may lead to sex reversal. For example, during the gonad development of blue tilapia (*Oreochromis aureus*, Steindachner, 1864), high water temperature could lead the gonads to differentiate into testes [5]. During the early gonad differentiation of channel catfish (*Ictalurus punctatus*, Rafinesque, 1818), high temperature will increase the proportion of females in the population [6]. Situations in which water temperature can lead to sex reversal have also been found in Japanese flounder (*Paralichthys olivaceus*, Temminck and Schlegel, 1846) [7], zebrafish (*Danio rerio*, Hamilton, 1822) [8,9,10], Nile tilapia (*Oreochromis niloticus*, Linnaeus, 1758) [11], Chinese tongue sole (*Cynoglossus semilaevis*, Günther, 1873) [12], and bluegill sunfish (*Lepomis macrochirus*, Rafinesque, 1819) [13]. Previous studies of sex reversal have mostly focused on sex differentiation-related genes and pathways. Nonetheless, how individuals perceive external temperature changes and alter the expression of critical genes associated with sex differentiation is still worth intensive studying.

Some temperature-sensitive genes that respond to temperature changes and produce a series of stress reactions have been reported. Among them, the highly conserved heat shock proteins (HSPs) exist in both prokaryotic and eukaryotic cells, and they are widely studied as key factors in the response to environmental pressure [14,15]. Especially, heat shock protein 70 kDa (*hsp70*) and heat shock cognate protein 70 kDa (*hsc70*), which both have high sequence homology and similar biochemical properties, play important roles in stress stimulation, immune response, cell proliferation, development, and death [14,16,17,18,19,20]. A role for HSPs in affecting the original genetic sex of organisms under temperature change has been found in many fish species. For example, in medaka (*Oryzias latipes*, Temminck and Schlegel, 1846), *hsp70.1* could directly regulate the expression of corticotropin-releasing hormone at high temperature and change the level of cortisol, thus inducing the sexual reversal [21]. In *D. rerio*, transcriptome analysis of the effect of high temperature at the gonad differentiation stage showed that *hsp90a*, *hsp70l*, and *hspbp1* could be candidate genes involved in the masculinization after heat shock treatment [22]. In *C. semilaevis*, transcriptome studies of heat treatment during the key period of sexual differentiation and heat shock at the end of sexual differentiation pointed to genes related to the HSP family, with *hsc70* being a key candidate gene for the formation of sex-reversed fish (pseudomale) [23,24]. Therefore, *hsc70* may contribute to the perception of environmental temperature, then subsequently affect sex differentiation.

*C. semilaevis* is broadly distributed in the coastal areas of China, and it is a vital economic fish for aquaculture. *C. semilaevis* has a female heterotypic sex determination system (ZW ♀/ZZ ♂) [25], and there is obvious sex dimorphism between males and females. The growth rate of the female is 2–4 times faster than that of male individuals, and the full length and weight of mature females can reach several times that of males [26]. In addition, the genetic sex of *C. semilaevis* can be overturned by environmental factors. At the early developmental stage, culture of *C. semilaevis* under normal temperature (22 °C) will cause approximately 14% of ZW genotypic females to undergo sex reversal, and in the high-temperature environment (28 °C), 73% of ZW genotypic females can become pseudomales (phenotypic male) [27]. Thus, *C. semilaevis* can be used as an appropriate model for studying the response to temperature changes in teleosts.

In the present research, we cloned and studied two genes of the HSP70 family, *hsc70* and *hsc70-like*. The relative quantification of the target genes in various tissues, different developmental stages, and under different heat treatments in *C. semilaevis* were certified. A dual-luciferase reporter assay coupled with a green fluorescent protein (GFP) reporter assay were used to test the abilities of *hsc70* and *hsc70-like* to respond to high temperature. Furthermore, an overexpression experiment was carried out in the testis cell line of *C. semilaevis* to examine the relationship between the target gene and sex related genes. Our findings suggested that both *hsc70* and *hsc70-like* respond to high temperature and play a major role in sex determination and differentiation in *C. semilaevis*.

## 2. Results

### 2.1. Cloning and Sequence Characteristics of hsc70 and hsc70-like

**Figure 1:**
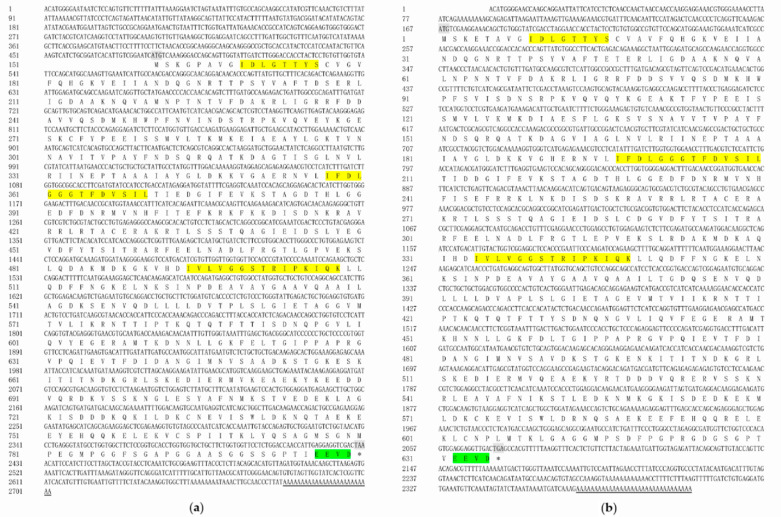
Full-length cDNA and predicted protein sequences of *C. semilaevis hsc70* and *hsc70-like* genes. (**a**) The mRNA and predicted protein sequence of *hsc70*; (**b**) The mRNA and predicted protein sequence of *hsc70-like*. The yellow shadows represented the conserved sequences of the HSP70 family, the green shaded EEVD is the cytoplasmic motif in the C-terminal region, the grey shadows represented the initiation codon and termination codon, and the asterisk (*) represented the termination codon. The poly (A) tail was underlined.

*hsc70* had a full-length sequence of 2702 bp, which included a 480 bp 5′UTR, 272 bp 3′UTR and 1950 bp ORF (GenBank accession number OQ225307). The sequence contained nine exons encoding 649 amino acids. The predicted protein had an estimated molecular weight of 71.00 kDa and a theoretical isoelectric point (pI) of 5.27 (Figure 1a). The full-length sequence of *hsc70-like* was 2396 bp, including a 166 bp 5′UTR, 322 bp 3′UTR, and 1908 bp ORF (GenBank accession number OQ225308). The sequence contained nine exons encoding 635 amino acids. The predicted protein sequence had a calculated molecular weight of 70.30 kDa and a theoretical isoelectric point (pI) of 5.35 (Figure 1b). Both *hsc70* and *hsc70-like* contained conserved sequences of the HSP70 family (IDLGTTYS, IFDLGGGTFDVSIL, IVLVGGSTRIPKIQK) and a conserved cytoplasm-specific EEVD sequence at the C-terminus (Figure 1).

### 2.2. Multiple Sequence Alignment and Phylogenetic Analysis

**Figure 2:**
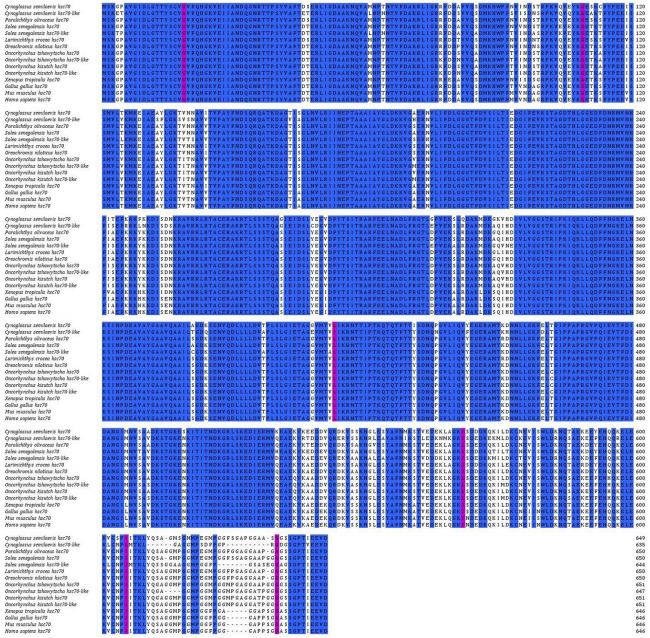
Multiple sequence alignment of HSC and HSC70-LIKE protein sequences from *C. semilaevis* and other vertebrates. GenBank accession numbers for protein sequences were as follows: XP_043872802.1 (*Solea senegalensis*), XP_020363711.1 (*Oncorhynchus kisutch*), XP_042153383.1 (*Oncorhynchus tshawytscha*), XP_019941072.1 (*Paralichthys olivaceus*), XP_043897279.1 (*Solea senegalensis*), XP_027129165.1 (*Larimichthys crocea*), XP_003448938.1 (*Oreochromis niloticus*), XP_024298732.1 (*Oncorhynchus tshawytscha*), XP_020358882.1 (*Oncorhynchus kisutch*), XP_002937574.2 (*Xenopus tropicalis*), NP_990334.2 (*Gallus gallus*), NP_112442.2 (*Mus musculus*), and XP_011541100.1 (*Homo sapiens*). The pink boxes indicated high similarity, while the blue boxes indicated completely identical residues.

**Figure 3:**
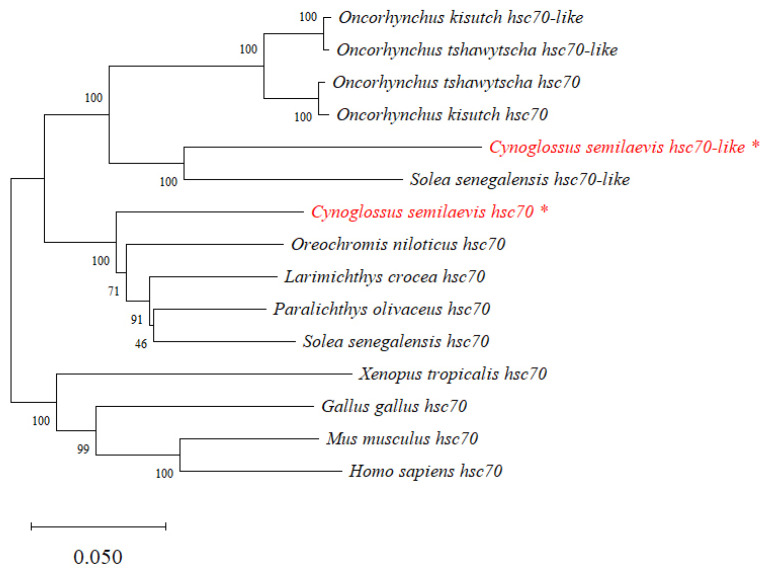
Phylogenetic tree of target genes. The tree was constructed using nucleic acid sequences of *hsc70* and *hsc70-like* from different species using the neighbor joining method with 1000 bootstrap replicates. Numbers at nodes indicated bootstrap support. *C. semilaevis hsc70* and *hsc70-like* genes were red and labelled with asterisk (*).

The protein sequences of HSC70 and HSC70-LIKE in *C. semilaevis* were aligned with sequences of HSC70 and HSC70-LIKE protein sequences in other teleost fishes, amphibians, birds, and mammals. The HSC70 and HSC70-LIKE protein sequences were highly similar in *C. semilaevis*, with significant differences found in the last 20 amino acids of the sequence. Moreover, these protein sequences showed high similarity up to 95% with that of other species (Figure 2). Due to the high similarity of protein sequences, a phylogenetic tree was constructed based on nucleic acid sequences. Figure 3 shows that known *hsc70* and *hsc70-like* form a homologous clade in teleosts, and *hsc70* forms another homologous clade in birds, amphibians, and mammals.

### 2.3. Expression Pattern of hsc70 and hsc70-like in C. semilaevis

**Figure 4:**
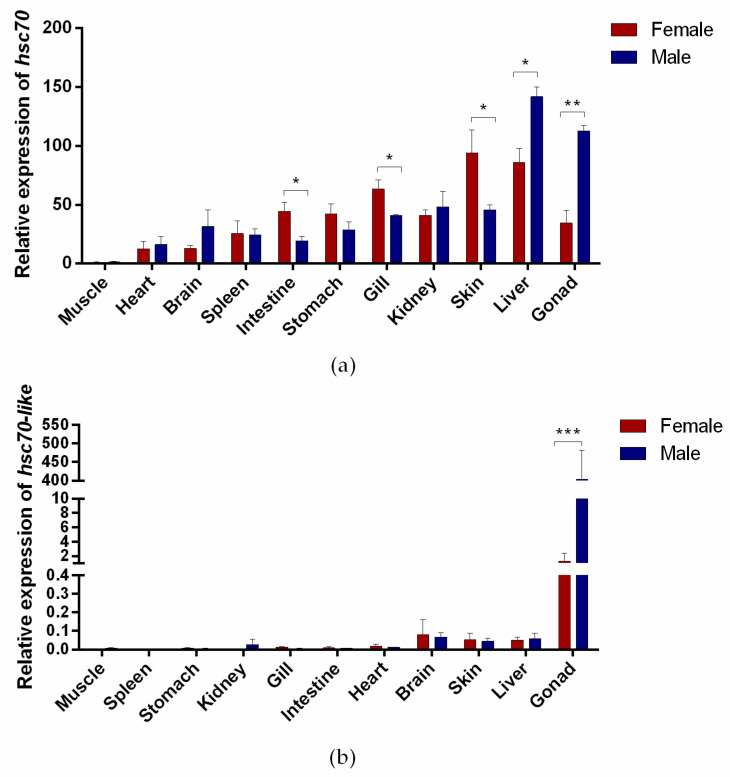
Expression of *hsc70* and *hsc70-like* genes in adult *C. semilaevis* tissues. (**a**,**b**) Detection of *hsc70* (**a**) and *hsc70-like* (**b**) expression in *C. semilaevis* tissues by RT—qPCR. Means ± SEM from three independent individuals (*n* = 3) were shown. *β-actin* was used as the reference gene. Asterisks indicated statistically significant differences (* *p* <0.05; ** *p* < 0.01; *** *p* < 0.001).

The expression of *hsc70* and *hsc70-like* genes in differ tissues of adult *C. semilaevis* was verified by RT—qPCR. The results showed that the expression of *hsc70* was enriched in gonads and much higher in testes than in ovaries. It also showed high expression in the heart, brain, spleen, intestine, stomach, gill, kidney, skin, and liver, and low expression only in the muscle (Figure 4a). The expression of *hsc70-like* was much higher in testes than that in ovaries, while its expression was hardly detected in the brain, skin, liver, muscle, spleen, stomach, kidney, gill, intestine, and heart (Figure 4b).

### 2.4. Expression Pattern of hsc70 and hsc70-like during Gonadal Development

**Figure 5:**
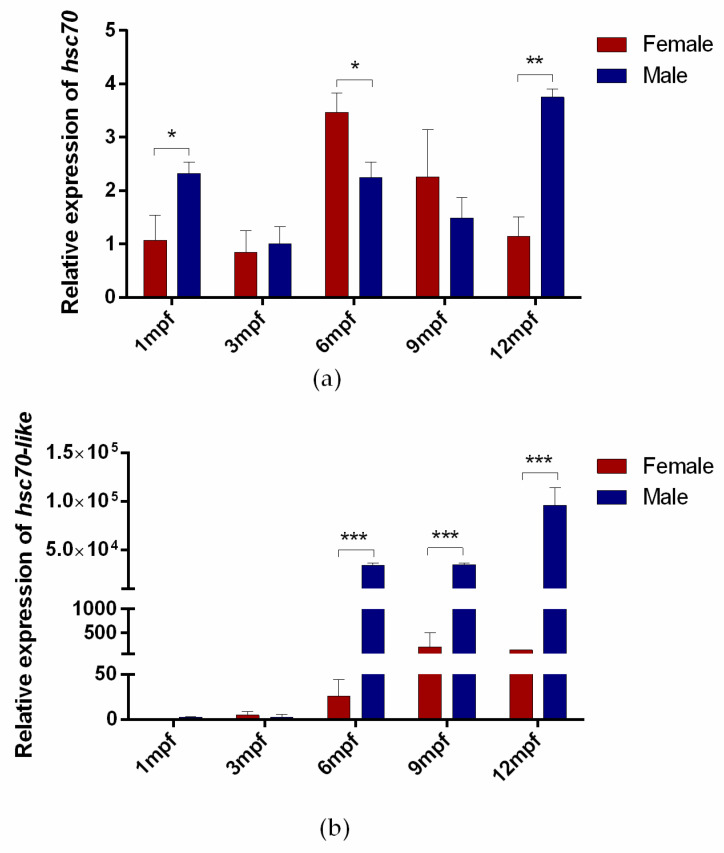
Expression of *hsc70* and *hsc70-like* genes in the gonads of male and female *C. semilaevis* at different stages. (**a**) Expression of *hsc70* during male and female gonadal development; (**b**) Relative gene expression of *hsc70-like* during male and female gonadal development. Relative mRNA levels were shown as the means ± SEM (*n* = 3), and values were normalized using *β-actin* as the internal control. * *p* <0.05; ** *p* < 0.01; *** *p* < 0.001. mpf, months post fertilization.

The expression levels of *hsc70* and *hsc70-like* genes in *C. semilaevis* during gonadal development were examined by RT—qPCR. The relative expression of *hsc70* showed statistically significant differences at 1 month post fertilization (1 mpf), 6 mpf, and 12 mpf between testes and ovaries in *C. semilaevis*, with high expression in testes at 1 mpf and 12 mpf and higher expression in ovary at 6 mpf (Figure 5a). *hsc70-like* still showed a testicular-higher expression pattern from 6 mpf on (Figure 5b).

### 2.5. Expression Patterns of hsc70 and hsc70-like in Gonads after Heat Treatment in C. semilaevis

**Figure 6:**
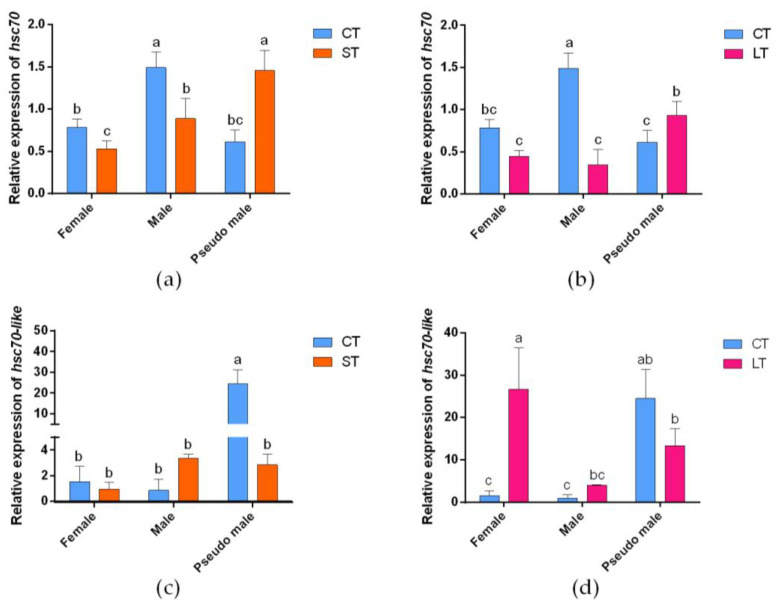
Gonadal expression of *hsc70* and *hsc70-like* genes in *C. semilaevis* under different high-temperature treatments. (**a**,**b**) Expression pattern of *hsc70* in the gonads of 3 mpf *C. semilaevis* under short-term (**a**) and long-term (**b**) high-temperature treatment; (**c**,**d**) Expression pattern of *hsc70-like* in the gonads of 3 mpf *C. semilaevis* under short-term (**c**) and long-term (**d**) high-temperature treatment. The expression of target genes were shown as means ± SEM (*n* = 3). *β-actin* as the internal control. CT, control treatment; ST, short-term heat stress treatment; LT, long-term heat treatment. Different letters indicated statistically significant differences (*p* < 0.05).

To verify whether the *hsc70* and *hsc70-like* genes could respond to high temperature, their relative expression levels were measured with RT—qPCR in the 3 mpf female, male, and pseudomale *C. semilaevis* under short-term and long-term 28 °C heat treatment. *hsc70* showed a similar expression trend in the *C. semilaevis* gonads in all sexes under these two modes of heat treatment, which was significantly downregulated in females and males while significantly upregulated in pseudomales (Figure 6a,b). On the other hand, *hsc70-like* showed significant downregulation under short-term heat stress and a downregulation trend of 45.6% under long-term heat treatment in pseudomales. There was a tendency for upregulation in males under these two modes of heat treatment, with the long-term increasing by 3.73 times and short-term increasing by 2.96 times. *hsc70-like* showed significant upregulation in females under long-term heat treatment (Figure 6c,d).

### 2.6. hsc70 and hsc70-like Genes Can Respond to High Temperature Rapidly In Vitro

**Figure 7:**
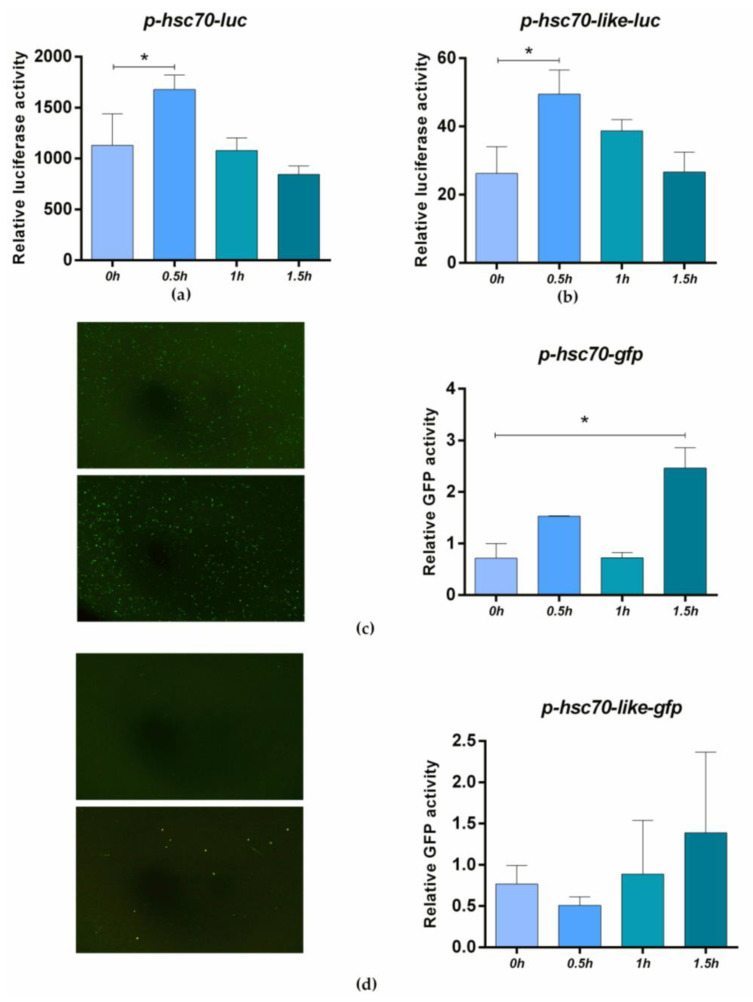
High temperature activates promoter activity of *hsc70* and *hsc70-like* genes *in vitro.* (**a**,**b**). Luciferase activity analysis of *hsc70*-promoter (**a**) /*hsc70-like*-promoter (**b**) under 42 °C heat shock in HEK293T cell and the pGL3-basic plasmid was used as a negative control; (**c**,**d**). *gfp* fluorescence analysis of *hsc70*-promoter (**c**) /*hsc70-like*-promoter (**d**) under 42 °C heat shock in HEK293T cell and the pEGFP-N3 plasmid was used as a negative control. The 42 °C heat shock treatment was conducted at 48 h post transfection. Luciferase activities and *gfp* relative expression were shown as means ± SEM (*n* = 3) and HEK293T *β-actin* was used as the internal reference (**a**–**d**). * *p* < 0.05.

To verify whether the *hsc70* and *hsc70-like* genes could respond to high temperature, a 2309 bp *hsc70* promoter and a 1781 bp *hsc70-like* promoter were cloned for activity analysis. The vectors p*hsc70*-luc, p*hsc70*-like-luc, p*hsc70*-*gfp*, and p*hsc70-like*-*gfp* were constructed. The dual-luciferase experiment showed that activity of both *hsc70* and *hsc70-like* promoter were significantly elevated by 1.49 times and 1.89 times, respectively, under 42 °C heat shock within 0.5 h compared to the control temperature (37 °C) (Figure 7a,b). Fluorescence observation and RT—qPCR analysis of *gfp* also confirmed that *hsc70* responded quickly to high temperature (Figure 7c,d).

### 2.7. hsc70 and hsc70-like Genes Can Affect the Expression of Sex-Related Genes under High Temperature

**Figure 8:**
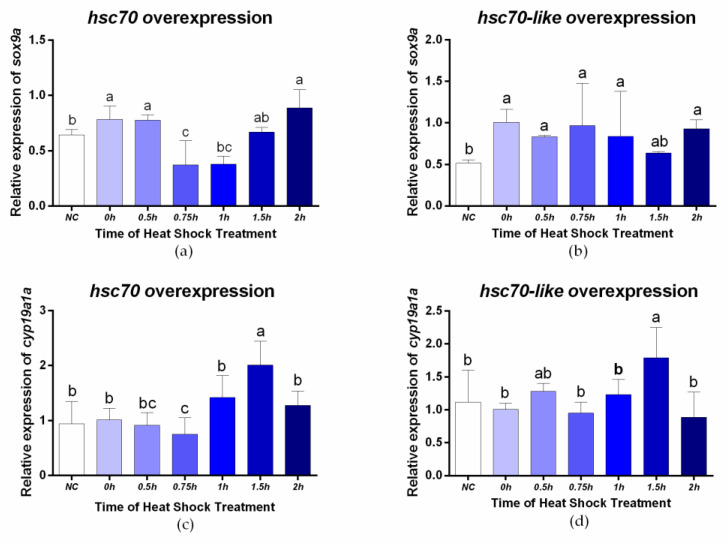
Analysis of sex-related genes after the overexpression of target genes under heat shock treatment. (**a**,**b**) Relative expression of *sox9a* in *C. semilaevis* testis cell line overexpression with *hsc70* (**a**) /*hsc70-like* (**b**); (**c**,**d**) Relative expression of *cyp19a1a* in *C. semilaevis* testis cell line overexpression with *hsc70* (**c**) /*hsc70-like* (**d**). After transfection with pcDNA3.1-*hsc70*/pcDNA3.1-*hsc70-like* for 24 h, 28 °C heat shock treatment was conducted and lasted for 2 h. pcDNA 3.1 plasmid was set as negative control (NC). Relative expression results were shown as the means ± SEM (*n* = 3), and *β-actin* was normalized as an internal standard. Different lowercase letters indicated statistically significant differences (*p* < 0.05).

To verify whether *hsc70* and *hsc70-like* were involved in sex determination and differentiation, the pcDNA3.1-*hsc70* and pcDNA3.1-*hsc70-like* were transfected into the testis cell line of *C. semilaevis*, followed by 28 °C heat shock treatment. The expression level of sex-related genes was detected by RT-qPCR. As shown in Figure 8a, overexpression of *hsc70* could significantly activate the male-related gene *sox9a*, while this activation was inhibited by high temperature, and the expression of *sox9a* decreased significantly from 0.75 h to 1 h post heat shock and rose back again from 1.5 h on. On the other hand, overexpression of *hsc70-like* could also significantly activate *sox9a*, and this activation was maintained under high temperature (Figure 8b). Neither *hsc70* nor *hsc70-like* overexpression had effect on the female-related gene *cyp19a1a* under normal temperature, while it increased *cyp19a1a* expression significantly at 1.5 h (Figure 8c,d).

## 3. Discussion

Our research verified the sequences of *hsc70* and *hsc70-lik*e genes from *C. semilaevis*. The HSC70 protein contained 649 amino acids, and the HSC70-LIKE protein contained 635 amino acids. Both of them contained three consensus-conserved sequences from the HSP70 family, as well as a conserved cytoplasmic-specific EEVD sequence at their C-terminus [28]. The HSC70 and HSC70-LIKE protein sequences differed significantly in the last 20 amino acids, and this region contained a low-complexity domain. Comparisons of the predicted sequences of HSC70 and HSC70-LIKE with similar proteins from various species showed a high identity. In the phylogenetic tree, the predicted target gene proteins were clustered into a class of HSC70 and HSC70-LIKE proteins in other teleosts. These results suggested that the HSC70 and HSC70-LIKE proteins were highly conserved structurally.

Studies have shown that there are many subtypes of HSC70 in an individual’s genome [29]. According to the expression analysis, *hsc70* was widely distributed in the tissues of 12 mpf *C. semilaevis* and only expressed at low levels in muscle. This extensive tissue distribution pattern has been verified in teleosts, for instance, rainbow trout (*Oncorhynchus mykiss*, Walbaum, 1792) [30], common carp (*Cyprinus Carpio*, Linnaeus, 1758) [31], and grass carp (*Ctenopharyngodon idella*, Valenciennes, 1844) [17]. This further supports that the *hsc70* sequence obtained in this experiment was correct. The expression pattern of *hsc70-like* was not exactly the same as that of *hsc70*. *hsc70-like* was specifically detected in the gonads, especially highly expressed in the testes. This differential expression pattern of different *hsc* genes has been previously reported [17,32,33]. At different stages of gonadal development, *hsc70-like* always showed a testicular-higher expression pattern from 6 mpf on, and *hsc70* exhibited ovarian-higher expression at only 6 mpf. Previous studies have shown that ovarian differentiation of *C. semilaevis* has been completed by 6 mpf [34], and oocytes began to proliferate and differentiate into the oogenesis stage [35]. It is inferred that *hsc70* may be vital in oogenesis, and *hsc70* and *hsc70-like* may be crucial in gonadal development.

To examine the relationship between the response of the *hsc70* and *hsc70-like* genes to external high temperature and the regulation of sex differentiation [24,27], we conducted different heat treatments during critical times of sex differentiation in *C. semilaevis*. The mRNA expression trend of *hsc70* was the same before and after short-term and long-term heat treatment (Figure 6a,b). However, *hsc70-like* showed different expression patterns under these two modes of heat treatment (Figure 6c,d). Therefore, it is speculated that both *hsc70* and *hsc70-like* may affect the sex of *C. semilaevis*, but their influence mechanisms may be different. This is possibly due to the significant differences between *hsc70* and *hsc70-like* in their carboxy-terminal domain, which is involved in mediating substrate specificity and specific biological functions [36]. In addition, the response of the target genes to high temperature was tested *in vitro*, and the results also showed that the target genes had a rapid response to high temperature. Under the action of the *hsc70* promoter and *hsc70-like* promoter, the expression of luciferase-reporter genes increased significantly after 0.5 h of thermal stress. It has been reported that the expression level of *hsc70* in tiger shrimp (*Penaeus monodon*, Fabricius, 1798) is also affected by temperature and could rapidly respond to high temperature *in vitro* [37]. Therefore, it is inferred that the rapid inductions of transcription for *hsc70* and *hsc70*-*like* may be relatively conserved across species.

After overexpression of the *hsc70* and *hsc70-like* genes in the *C. semilaevis* testis cell line and heat shock treatment, it was further proven that the target genes can affect the relative mRNA level of the sex-related genes *sox9a* and *cyp19a1a* [38,39,40,41,42]. Figure 8 shows that overexpression of *hsc70* and *hsc70*-*like* could directly activate the expression of *sox9a* and that *sox9a* expression changed with the length of heat shock treatment time until it remained at a high expression level. In the high-temperature experiment *in vivo*, the results showed that the expression level of *hsc70* in pseudomales and females was comparable in the normal-temperature group, while high temperature significantly upregulated the expression level of *hsc70* in pseudomales to approach the expression level of males at normal temperature. The expression of *hsc70*-*like* was highest in pseudomales at normal temperature, while significantly down-regulated at high temperature. It is speculated that *hsc70* and *hsc70*-*like* may be noteworthy in the process of sex reversal induced by high temperature, and that these two genes seem to have a coordinated effect in pseudomales. At normal-temperature, the *hsc70-like* gene was responsible for elevating the expression of male-related genes such as *sox9a*. While at high temperature, *hsc70* gene switched to rapidly upregulated and participate in promoting male-related genes expression. The overexpression of *hsc70* and *hsc70*-*like* cannot directly affect the expression of *cyp19a1a*, but it will affect the response of *cyp19a1a* to high temperature.In females, long-term high temperature inhibits the expression of *hsc70* expression and activates the expression of *hsc70-like*, thus keep the high expression of female sex-related genes such as *cyp19a1a* to maintain female sexual characteristics. In pseudomales with phenotypic males, high temperature inhibits the expression of *hsc70*-*like* and promote the expression of *hsc70*, which may inhibit *cyp19a1a* and promoting *sox9a*, resulting in the production of pseudomales. Hence, these two genes may be potential molecular markers for identifying pseudomales. However, since samples during the formation process of pseudomale fish were hardly collected, the assumption is still need to be verified.

## 4. Materials and Methods

### 4.1. Ethics Statement

All *C. semilaevis* samples were obtained from the Haiyang High-Tech Experimental Base (Haiyang, China) and acclimated for 48 h under laboratory conditions. After approval by the Institutional Animal Care and Use Committee (IACUC) of the Yellow Sea Fisheries Research Institute (CAFS) (Qingdao, China), the fish were collected and handled in accordance with the “Guidelines for Experimental Animals” of the Ministry of Science and Technology (Beijing, China).

### 4.2. Heat Shock Treatment Experiment and Fish Collection

Female and male samples of juvenile and adult fish reared under normal temperature (22 °C), at 1 mpf, 3 mpf, 6 mpf, 9 mpf, and 12 mpf were collected. Eleven tissue samples were collected for 12 mpf *C. semilaevis*, including heart, brain, liver, spleen, kidney, intestine, stomach, gill, skin, muscle, and gonads. Samples of gonad were taken from the remaining four stages, as well as from the 3 mpf females, males, and pseudomales after high-temperature (28 °C) long-term heat treatment (from 1 mpf to 3 mpf) and 48 h short-term heat treatment. Triplicate samples were frozen in liquid nitrogen and then stored in a freezer at −80 °C until use. The tail fin of each fish was soaked in 75% ethanol for extraction genomic DNA.

### 4.3. Extraction of Total RNA/Genomic DNA, and Identification of Genetic Sex

TRIzol reagent (Invitrogen, Carlsbad, CA, USA) was used to extract total RNA from each sample. RNA concentration was measured with a NanoDrop 2000 spectrophotometer (Thermo, Waltham, MA, USA), and quality was assessed with agarose gel electrophoresis. A traditional phenol-chloroform extraction method was used to obtain genomic DNA from the tail fin. Using established methods and primer pairs (sex F and sex R in Table 1), we determined genetic sex of each fish.

### 4.4. Obtaining the Full-Length cDNA of hsc70 and hsc70-like though RACE

The first strand cDNA was synthesized from purified total RNA using the PrimeScriptTM II 1st Strand cDNA Synthesis Kit (Takara, Kusatsu, Japan). Primers (Table 1) were designed based on predicted sequences in the NCBI database to obtain partial cDNA fragments of *hsc70* and *hsc70-like* with PCR. After sequence confirmation of the CDS region, 5′ and 3′ gene-specific primers (GSPs) and nested gene-specific primers (NGSPs) were designed for 5′ and 3′ Rapid Amplifi-cation of cDNA Ends (RACE) PCR (Table 1). In accordance with the manufacturer’s instructions, we used the SMARTer^®^ RACE 5’/3’ Kit (Clontech, Mountain View, CA, USA) to amplify the 5′ and 3′ ends of *hsc70* and *hsc70-like*. The PCR products were detected by agarose gel electrophoresis, and the corresponding band of interest was purified using a product purification kit (Vazyme, Nanjing, China). Finally, the purified fragments were connected to the pEASY-T1 (TransGen, Beijing, China) vector for Sanger sequencing.

### 4.5. Bioinformatics Analysis

A BLASTn tool (http://blast.ncbi.nlm.nih.gov/Blast.cgi (accessed on 27 March 2022) was used to confirm the open reading frame (ORF). Expasy (https://prosite.expasy.org/ (accessed on 3 April 2022) was used to predict the protein domains. Multiple alignment of protein sequences from teleost fishes, amphibians, birds, and mammals were performed with Clustal Omega (http://www.clustal.org/omega/ (accessed on 5 April 2022) and colored using Adobe Illustrator 2020 (Adobe, San Jose, CA, USA). A neighbor-joining phylogenetic tree with 1000 bootstraps was constructed in MEGA-X.

### 4.6. Real-Time Quantitative PCR

TRIzol-extracted total RNA was synthesized into cDNA templates by using the PrimeScript RT Reagent Kit with gDNA Eraser (Takara, Dalian, China). The QuantiNova SYBR Green PCR Kit (Qiagen, Hilden, Germany) was used for the RT—qPCR experiments. The system was 10 μL, which contained 5 μL 2 × SYBR Green PCR Master Mix, 0.4 μL primers (Table 1), and 1 μL of cDNA. Cycles consisted of 95 °C for 2 min, followed by 40 cycles of 95 °C for 5 s, and 60 °C for 10 s. Melting curve reaction conditions were 95°C for 15 s, 65 °C for 1 min, +0.11 °C/s to 95 °C, and 40 °C for 10 s, using a LightCycler ^®^ 480 II system (Basel, Switzerland). *β-actin* was used as an internal reference gene. Each experiment was performed with three biological and three technical replicates. The data were processed by the 2^−ΔΔCt^ method, and the relative expression of the target genes were analyzed by GraphPad Prism 6.0 (GraphPad, San Diego, CA, USA). Finally, *t*-tests or one-way ANOVA were used to ascertain significant differences.

### 4.7. Promoter Cloning and Plasmid Construction

Based on the *C. semilaevis* genomic sequencing data, the primer pair *hsc70*-pro F/R was designed for amplification and validation of the 2312 bp 5’ upstream sequence of *hsc70* promoter, with *Sac*I and *Xho*I recognition sites added to the 5’ ends of each specific primer, respectively (Table 1). The primer pair *hsc70-like*-pro F/R was designed to amplify and verify the 1781 bp 5’ upstream sequence of *hsc70-like* promoter. For forward and reverse primers, *Kpn*I and *Xho*I identification sites were added at the 5’ ends, respectively (Table 1). The purified PCR product and pGL3-basic vector (Promega, Madison, WI, USA), which contains the firefly luciferase gene, were cleaved with corresponding enzymes and ligated with T4 DNA ligase (Monad, Suzhou, China) to obtain p*hsc70*-luc and p*hsc70-like*-luc. Negative control was set with pGL3-basic, and positive control was set with pGL3-control. For the GFP observation experiment, *Nde*I and *Xho*I identification sites were added to the 5’ ends of each promoter primer, respectively (Table 1). The purified PCR product and pEGFP-N3 vector, which contains the GFP, were cleaved with corresponding enzymes and ligated with T4 DNA ligase to obtain p*hsc70*-*gfp* and p*hsc70-like*-*gfp*. The pEGFP-N3 vector was used as the negative control. The ORFs of *hsc70* and *hsc70-like* were amplified and cloned into the *Kpn*I and *Xba*I identification sites of pcDNA3.1(+) (Invitrogen) to obtain pcDNA3.1-*hsc70* and pcDNA3.1-*hsc70-like*. An EndoFree Plasmid Mini Kit (TIANGEN, Beijing, China) was used to prepare all plasmids.

### 4.8. HEK293T Cell Culture, Transfection, Heat Shock, and Luciferase Assay

Human embryonal kidney (HEK) 293T cells used in the study was purchased from the Shanghai Institute of Cell Biology and were cultured using 293T [HEK-293T] cell-specific medium (Procell, Wuhan, China). The incubating conditions were 37 °C with 5% CO_2_. For the dual-luciferase experiment, 500 ng of promoter-luciferase overexpression vector and 40 ng of pRL-TK plasmid (Renilla luciferase gene driven by HSV thymidine kinase promoter) were cotransfected in a 24-well culture plate using Lipofectamine 3000 Reagent (Invitrogen). After cotransfection for 48 h, the heat treatment was conducted with a water bath that gradually increased the temperature from 37 to 42 °C. The heat shock was conducted for 0.5, 1, and 1.5 h, respectively. Cells were collected using the Dual-Luciferase ^®^ Reporter Assay System (Promega) and Varioskan Flash Spectro-Scopic Scan multimode reader (Thermo, Vantaa, Finland) for testing. For the GFP reporter assay, 500 ng of promoter-*gfp* vector was transfected in a 24-well culture plate using Lipofectamine 3000 Reagent. After 48 h of transfection, the same heat treatment was performed as in the dual-luciferase experiment. The *gfp* fluorescence was observed and recorded using an Olympus IX73 inverted microscope (Olympus, Tokyo, Japan). Each experiment was performed in triplicate.

### 4.9. Culture, Transfection, and Heat Shock of C. semilaevis Testis Cell Line

The *C. semilaevis* testis cell line originated from the researchers’ own laboratory and was cultured at 24 °C in L15 medium (Solarbio, Beijing, China) added with 20% fetal bovine serum (FBS) (Gibco, Carlsbad, CA, USA), 2 ng/mL bFGF, 2 ng/mL LIF, 800 U/mL penicillin, 800 μg/mL streptomycin, and 400 μg/mL gentamicin (Solarbio). Lipofectamine 3000 Reagent was used to transfect 500 ng of pcDNA 3.1-*hsc70* and pcDNA 3.1-*hsc70-like* into 24-well plates. After transfection for 24 h, the cells were treated in a water bath gradually heated from 24 to 28 °C and heat treated for 0.5, 0.75, 1, 1.5, and 2 h, respectively. RNA was extracted using TRIzol reagents and sex-related gene expression abundance was detected with RT—qPCR. Three repetitions were performed each treatment.

### 4.10. Statistical Analysis

All experiments were performed in triplicate. All data were shown as means ± SEM. Prism 6.0 (GraphPad) was used for data processing, and *t*-tests and one-way ANOVA followed by Bonferroni’s multiple comparison tests were used for analysis; *p*-values of ≤0.05 were considered statistically significant. (* *p* < 0.05; ** *p* < 0.01; *** *p* < 0.001).

## 5. Conclusions

In conclusion, the *hsc70* and *hsc70-like* genes were validated in *C. semilaevis* whose sexes can be related to ambient temperature. According to bioinformatics analysis, the *hsc70* and *hsc70-like* genes were highly conserved in vertebrates. In addition, the expression pattern results showed that both *hsc70* and *hsc70-like* were highly expressed in the gonads of adult fish and much higher in testes than in ovaries. At different stages of gonadal development, *hsc70* showed ovarian-higher expression at 6 mpf, and *hsc70-like* showed a testicular-higher expression from 6 mpf on. All these findings supported the potential role of the target genes in sex determination and differentiation. Both long-term heat treatment during the temperature-sensitive sex-determining period and short-term heat stress at the end of this period caused upregulated expression of *hsc70* in pseudomales and downregulated expression of *hsc70-like* in pseudomales. The dual-luciferase assay and fluorescence observation also proved that the target genes could rapidly respond to high temperature *in vitro*. Heat shock treatment of *C. semilaevis* testis cell line overexpressed with *hsc70* and *hsc70-like* could affect the expression of sex-related genes *sox9a* and *cyp19a1a*. These results indicated that *hsc70* and *hsc70-like* could rapidly respond to external high temperatures and were related to gonadal differentiation. Therefore, *hsc70* and *hsc70-like* are key elements linking external high temperature signals with sex differentiation *in vivo*.

## Figures and Tables

**Table 1:** Primers used in this report.

Primer Name	Sequence (5’-3’)	Purpose
*hsc70*-F	CGAAGCATGTAACTTCCTTTTC	Partial fragment
*hsc70*-R	GTGATGAACCGAGTGATACCAA	amplification
*hsc70-like*-F	TCATCCTCTCAACCAACTAACC	
*hsc70-like*-R	TCACATCCTCACAGATCAAAAA	
*hsc70*-pro-F	GAAAGATACAGTTAAAGCCCCAGG	
*hsc70*-pro-R	CATGATTCCTAGTTAGAAAAAGAAAAGTG	
*hsc70-like*-pro-F	TCCATTAGAAGCCCCTCACGAT	
*hsc70-like*-pro-R	GTCTTTGAACCTGAGGAGGCAAC	
*hsc70*-5’gsp	TGTTGTCCTGTTGCCCTGGTCGTTGGC	RACE
*hsc70*-3’gsp	GGTATGCCTGGTGGCTTCTCCGGTGCA	
*hsc70*-5’ngsp	CGGTGTTAGAAGGAAAAGGAAG	
*hsc70*-3’ngsp	TCTGGACCAACCATTGAGGAGGTCGAC	
*hsc70-like*-5’gsp	CATGCTGGAACACGGCCACACAGGA	
*hsc70-like*-3’gsp	CTGATTTCCCTGGGCCTAGAGGCGA	
*hsc70-like*-5’ngsp	TTCGACATGTCTTTGAACCTGAGGGGTT	
*hsc70-like*-3’ngsp	ACAGCAGTTGTACCAGTTCACAGACGTT	
*hsc70*-qF	ACAAGAACCAGACTGCCGAGAAG	RT—qPCR
*hsc70*-qR	CCTCAGGCATGTTACCAGACATTC	
*hsc70-like*-qF	AGTGTGTCAAACGCCGTGGTAA	
*hsc70-like*-qR	CCACCTTTTTGTCCAGACCGTAG	
*β-actin*-qF	GCTGTGCTGTCCCTGTA	
*β-actin*-qR	GAGTAGCCACGCTCTGTC	
*gfp*-qF	ACAACAGCCACAACGTCTAT	
*gfp* -qR	ATGTTGTGGCGGATCTTGAA	
293T*β-actin*-qF	GATGATATCGCCGCGCTCGT	
293T*β-actin*-qR	GTAGATGGGCACAGTGTGGGTG	
*sox9a*-qF	CAGGCAGGTAATGTTGGGGT	
*sox9a*-qR	AAGGAGCCGTAGGTGATGTG	
*cyp19a1a*-qF	CTCTGTTCCTCAGGTTTCTCTC	
*cyp19a1a*-qR	GATGTGACCCAGTGTGTGTTG	
*hsc70*-proluc-F	cgagctcGAAAGATACAGTTAAAGCC	Plasmid construction
*hsc70*-proluc-R	ccgctcgagGATTCCTAGTTAGAAAAAG	
*hsc70*-progfp-F	ggaattccatatgGAAAGATACAGTTAAAGCCCCAG	
*hsc70*-prgfp-R	ccgctcgagGATTCCTAGTTAGAAAAAGAAAAGTGAT	
*hsc70-like*-proluc-F	ggggtaccTCCATTAGAAGCCCCTCAC	
*hsc70-like*-proluc-R	ccgctcgagGTCTTTGAACCTGAGGA	
*hsc70-like*-progfp-F	ggaattccatatgTCCATTAGAAGCCCCTCAC	
*hsc70-like*-progfp-R	ccgctcgagGTCTTTGAACCTGAG	
*hsc70*-CDS-F	ggggtaccATGTCAAAGGGACCAG	
*hsc70*-CDS-R	gctctagaGTCGACCTCCTCAATGGTTG	
*hsc70-like*-CDS-F	ggggtaccATGTCGAAGGAAACAGC	
*hsc70-like*-CDS-R	gctctagaGTCAACCTCCTCCACTGTG	
sex-F	CCTAAATGATGGATGTAGATTCTGTC	Sex identification
sex-R	GATCCAGAGAAAATAAACCCAGG	

The primer sequence is indicated by uppercase letters, while restriction enzyme sites with protective nucleotides are indicated by lowercase letters.

## Data Availability

Data is contained within the article.
